# Chemical Composition and Potential Environmental Impacts of Water-Soluble Polar Crude Oil Components Inferred from ESI FT-ICR MS

**DOI:** 10.1371/journal.pone.0136376

**Published:** 2015-09-01

**Authors:** Yina Liu, Elizabeth B. Kujawinski

**Affiliations:** Department of Marine Chemistry & Geochemistry, Woods Hole Oceanographic Institution, Woods Hole, Massachusetts, 02543, United States of America; Texas A&M University at Galveston, UNITED STATES

## Abstract

Polar petroleum components enter marine environments through oil spills and natural seepages each year. Lately, they are receiving increased attention due to their potential toxicity to marine organisms and persistence in the environment. We conducted a laboratory experiment and employed state-of-the-art Fourier-transform ion cyclotron resonance mass spectrometry (FT-ICR-MS) to characterize the polar petroleum components within two operationally-defined seawater fractions: the water-soluble fraction (WSF), which includes only water-soluble molecules, and the water-accommodated fraction (WAF), which includes WSF and microscopic oil droplets. Our results show that compounds with higher heteroatom (N, S, O) to carbon ratios (NSO:C) than the parent oil were selectively partitioned into seawater in both fractions, reflecting the influence of polarity on aqueous solubility. WAF and WSF were compositionally distinct, with unique distributions of compounds across a range of hydrophobicity. These compositional differences will likely result in disparate impacts on environmental health and organismal toxicity, and thus highlight the need to distinguish between these often-interchangeable terminologies in toxicology studies. We use an empirical model to estimate hydrophobicity character for individual molecules within these complex mixtures and provide an estimate of the potential environmental impacts of different crude oil components.

## Introduction

Each year, millions of barrels of oil are released to the global ocean from natural and anthropogenic sources [1]. For example, in 2010, the Deepwater Horizon (DWH) oil spill released ~5 million barrels of oil and gas from the Macondo well to the Gulf of Mexico [2]. Although on a smaller scale, the West Falmouth oil spill in 1969 resulted in petroleum contaminants that persisted for decades in the sediments [3]. Oil spills threaten global and local environments over both short and long time scales. Consequently, the fate and environmental impacts of crude oil and refined petroleum products merit significant investigation [4–9]. Most of these studies focus on the major components in crude oil, i.e. the non-polar hydrocarbons. These compounds contain only carbon and hydrogen and have been studied extensively with gas chromatography (GC)-based analytical methods. These methods cannot resolve the minor fraction of crude oil that contains heteroatoms such as nitrogen (N), sulfur (S), and oxygen (O). However, these compounds may be disproportionately important for ecosystem health and recent studies have urged the investigation of non-weathered and weathered oil components that contain N, S, and O [5, 8, 10, 11].

Reddy et al. [5] showed that the traditional molecular-level methods afforded by conventional GC characterized only half of the fluid released from the Macondo well during the DWH incident. Polar compounds, which are defined as compounds that contain one or more heteroatoms (NSO) [12, 13], may account for a considerable fraction of the uncharacterized materials. Following the 2007 M/V Cosco Busan spill in San Francisco Bay, Incardona et al. [10] showed that the unexpectedly high mortality rate of herring spawn in oiled intertidal zones could be attributed to the presence of unidentified compounds derived from the weathered bunker oil. Their results are consistent with a recent study by Aeppli et al. [8], which showed that oxygenated residues are formed during weathering and that these residues are likely toxic and not amenable to GC analysis. Currently, ultrahigh resolution mass spectrometry, or Fourier-transform ion cyclotron resonance mass spectrometry (FT-ICR-MS), is the only method that allows molecular-level characterization of polar (*i*.*e*. NSO-containing) crude oil derived compounds [12–14].

FT-ICR-MS offers ultrahigh mass resolving power greater than 100,000 with high mass accuracy (< 1 ppm uncertainty) [13, 15, 16], and thus, is capable of resolving tens of compounds per nominal mass [17]. When coupled to ionization sources such as electrospray ionization (ESI), atmospheric pressure photoionization (APPI) and chemical ionization (APCI), these analyses provide unprecedented views of the chemical diversity of polar and non-polar components of complex organic mixtures such as natural organic matter and crude oils [13–16, 18–21]. With the superb mass resolving power, it is possible to accurately assign elemental formulas to a substantial percentage (~90–95%) of the detected masses, and hence this technique has enabled researchers to gain molecular level insights into these complex mixtures [17]. It should be noted, however, that data generated with FT-ICR-MS are only semi-quantitative due to ion suppression of certain chemical species. Consequently, the detected peak intensities cannot be converted to absolute concentrations [16]. Nonetheless, this state-of-the-art analytical method is widely employed in the field of petroleum characterization, particularly for the identification of NSO-containing compounds [12–14].

Despite their minor proportion (< 15% by mass) in crude oils [12], NSO-containing compounds provide geochemical insights to the crude oil sources, but more importantly, have been implicated in oil refinement complications due to their corrosive properties [12]. The Macondo oil is a light sweet crude oil with a trace amount of heteroatom-containing compounds [14], while polar compounds often constitute a considerable fraction of immature or weathered crude oils [22]. Within the polar fraction, the relative proportions of different heteroatom classes also vary with the type of crude oil [12, 13]. Recently, FT-ICR-MS analysis provided the first chemical “fingerprint” of the Macondo oil, and revealed that the most abundant compound classes in the bulk crude oil contain only one or zero heteroatoms [14]. Nevertheless, it has been suggested that polar compounds can account for ~70% of the petroleum-derived components dissolved in water [23], and that the most abundant compound classes in crude oil-derived water-soluble fractions often contain multiple heteroatoms [11, 24]. Polar crude-oil derived compounds may be more toxic to marine organisms than non-polar hydrocarbons [25] and are potentially more persistent in the environment [14, 26]. The DWH incident was the largest offshore oil spill in the U.S. history [4]. It is, therefore, crucial to understand the chemical and ecological impacts of the water-soluble polar compounds derived from the Macondo oil on the marine environment.

The toxicity of water-soluble components derived from crude and refined oils to aquatic organisms were recognized as early as the 1970s [27–29]. Since then, there have been extensive discussions of the chemical distinctions between the different operationally-defined fractions containing dissolved crude or refined oil components, i.e. “water-accommodated fraction” (WAF) and “water-soluble fraction” (WSF) [27, 30–32]. By definition, WAF is the aqueous phase after fresh or saline water is mixed with crude oils for a defined period under either low or high energy, and thus contains variable amounts of oil droplets [33]. WSF is a subset of WAF, produced either by filtration through defined pore sizes or by centrifugation to remove oil droplets. The goal of a WSF preparation is the inclusion of only truly soluble compounds [30, 31, 33, 34]. There are multiple methods that can be used to generate WAF [34–36], and the need to have a consistent method for oil-spill investigations was suggested in 2000 [34]. However, to date, the scientific community has not reached a consensus for preparation of either WAF or WSF and for distinguishing between the chemical and toxicological characteristics of each fraction. Instead, the terms WAF and WSF have been used interchangeably in studies involving dissolved petroleum hydrocarbons [37–40].

Singer et al. [34] proposed that “water-accommodated” is the more accurate terminology, as there are often no subsequent treatments to remove microscopic oil droplets and fine particles as well as partially dissolved compounds from the aqueous phase. Filtration is avoided in some cases for it poses problems such as adsorption of compounds of interest [34] and variability in filtrate composition due to choice of filter pore size and material [30]. For instance, cellulose acetate filters retain more petroleum hydrocarbons than glass fiber filters, and smaller filter pore sizes substantially reduce the hydrocarbon concentrations in the filtrate [30]. Regardless, depending on the method used to create WAF, the composition of WAF and WSF may be drastically different. For example, the composition of WAF created from high energy mixing may more closely resemble the parent oil [35, 41], as the vigorous mixing could cause emulsification [34]. Variability in sample preparation, therefore, can lead to substantial differences in chemical or toxicity assessments. For example, Carls et al. [42] reported that the dissolved crude oil fraction was responsible for fish embryo damage, and that the inclusion of oil droplets may have led to an underestimation of toxicity of the dissolved compounds.

Information on the chemical fingerprints of polar water-soluble crude oil compounds is scarce. In an ideal solution, crude oil dissolution in water should be primarily controlled by factors such as solute molecular size and polarity (i.e. heteroatom content) as well as temperature, pressure, and the ionic strength of the solution. In natural seawater, however, factors that affect crude oil dissolution are more complex. Seawater contains thousands of organic molecules that can interact with petroleum hydrocarbons and hence affect their solubility [30, 31, 43]. An earlier study showed that the composition of dissolved organic matter (DOM) in seawater affects the amount and type of dissolved petroleum hydrocarbons [30], which could in turn influence their toxicity to marine organisms [31]. In the present study, we conducted a crude oil dissolution experiment with coastal seawater from Vineyard Sound, MA to (1) investigate the general compositional characteristics of water-soluble polar crude oil components in comparison with the parent oil and background DOM; (2) assess the factors that govern partitioning of these polar compounds into seawater; (3) investigate the chemical compositional differences between WAF and WSF; and (4) estimate the potential environmental impact of the polar compounds based on their chemical characteristics.

## Materials and Methods

### Experimental Setup

Vineyard Sound seawater (VSW) was collected at a public beach at Woods Hole, MA (specific sampling permission was not required). Marlin platform crude oil (Lot# SO-20111116-MPDF-003) was used as a surrogate for the Macondo oil in this dissolution experiment.

VSW was filter-sterilized through 0.2-μm Omnipore membrane filters (Millipore). Filter-sterilization does not completely remove bacteria from seawater, and thus bacterial contamination during the mixing of crude oil and seawater was possible. Autoclaving, however, was avoided to limit chemical alteration of the background DOM. It has been shown that microbial degradation of hydrocarbons during the DWH incident occurred in succession, with microbes degraded small hydrocarbons (<100 *m/z*) and *n*-alkanes enriched first in the early stage of oil degradation in the subsurface plume [44–46]. Therefore, the possibility of bacterial contamination should not affect our results and data interpretation given the mass range in our analytical window (140 to 1000 *m/z*).

To examine the effect of background lipophilic DOM on crude oil solubility, an aliquot of VSW was extracted with dichloromethane (DCM) to remove lipids and similar compounds (VSWE). Five mL of crude oil was added to the surface of 95 mL VSW and VSWE and mixed for 7 days in the dark at room temperature under low energy, i.e. no visible vortex formed in the water-column, to ensure no visible oil droplets were entrained into the water phase [11, 34]. VSW and VSWE controls (no oil added) were kept under the same conditions (sample list in S1 Table). All solvents used in this study were Optima-grade or better and all glassware was acid-cleaned and combusted at 450°C for at least 4 hours.

### Water-Accommodated vs. Water-Soluble Crude Oil Fractions

We examined two operationally-defined aqueous fractions of petroleum compounds in our experiments, the WAF and the WSF. Here, we explicitly distinguished the two fractions as follows: after 7 days of mixing, the crude oil layer was removed with combusted glass pipets from each sample. The resulting aqueous phase is the WAF, and contains (1) trace amounts of dispersed microscopic oil droplets and fine particles at the oil-seawater interface; (2) partially dissolved (i.e. semi-soluble) compounds; and (3) water-soluble compounds. A replicate of WAF from each treatment was filtered through a combusted 0.7 μm GF/F glass fiber filter (Whatman) to obtain the operationally-defined dissolved fraction, or WSF.

### Sample Extraction

Three fractions were collected for each aqueous sample and seawater control following established protocols [47]. Briefly, each sample was extracted three times with DCM (1:3 v:v); the combined extract is hereafter referred to as DCM 1. Subsequently, the aqueous phase was acidified to pH ~3 with concentrated hydrochloric acid (HCl) and extracted with DCM three times (pooled extract = DCM 2). Lastly, the aqueous phase was passed through PPL solid phase extraction resin and eluted with 100% methanol (MeOH) following established protocols [48] (extract = DCM-PPL). The two DCM extracts, DCM 1 and DCM 2, should contain lipophilic compounds that are neutral and/or weakly acidic and acidic, respectively. Lipophilic is used here to describe compounds removed from aqueous samples by dichloromethane extraction, rather than exclusively compounds that function as lipids in biological systems. The DCM-PPL represents the most hydrophilic compounds.

Pooled dichloromethane (DCM) extracts (DCM 1 and DCM 2, ~300 mL) were dried with combusted sodium sulfate (Na_2_SO_4_). Solvent in each sample was removed using the rotary evaporator to a volume of ~0.5 mL, diluted with 1–2 mL DCM, and then transferred to an 8 mL amber vial. The samples in DCM were then blown to dryness under gentle streams of ultrapure nitrogen gas (N_2_). The dried samples were stored at -20°C until analysis. The PPL solid phase extracted samples (DCM-PPL and direct PPL extract of seawater controls; see S1 Table) were stored in methanol (MeOH) at -20°C.

On the day of analysis, DCM extracted samples were reconstituted with 1 mL 50:50 toluene/MeOH with 1% NH_4_OH (added to promote negative ion formation in a relatively non-polar solvent). MeOH in the PPL solid phase extracted samples were removed by vacufuge (Eppendorf) to a volume of <10 μL then diluted with 1 mL 50:50 water/MeOH. The crude oil control was created by diluting oil with 50:50 toluene/MeOH with 1% NH_4_OH to achieve a final concentration of 6 mg mL^-1^.

### FT-ICR-MS Analysis

All samples were analyzed on a hybrid 7.0 T linear ion trap (LTQ) Fourier transform ion cyclotron mass spectrometer (FT-ICR-MS; LTQ FT Ultra, Thermo Scientific) coupled with electrospray ionization negative mode (ESI (-)) at 400,000 resolving power (*m/Δm*_*50%*_ at *m/z* 400) [49]. The instrument was externally calibrated to a mass accuracy of < 2 ppm with a standard solution from Thermo Fisher Scientific. Samples were directly infused into the ESI source at a flow rate of 4 μL min^-1^. The ESI spray voltages were between 3.2 and 3.5 kV. The capillary temperature was 235°C and 265°C for DCM-extracted and PPL-extracted samples, respectively. A sweep gas flow of 1 arbitrary unit was applied when analyzing the crude oil control. Data were acquired using the Xcalibur 2.0 package in conventional full scan mode at 140 to 1000 *m/z* and 200 to 1000 *m/z* for DCM-extracted and PPL-extracted samples, respectively. At least 200 individual transients were collected for each sample. Transients were co-added, Hanning apodized, zero-filled once, and then Fourier transformed using the MATLAB code provided by Southam et al [50]. Peaks with a signal-to-noise ratio above 5 were retained, where the noise is defined as the root mean square (RMS) calculated for a signal-free region of the spectra [50]. Peaks were internally re-calibrated using a list of *m/z* values present in a majority of the samples and with similar mass error [51]. Elemental formulas were assigned to aligned peaks using the Compound Identification Algorithm (CIA) [17, 20]. CIA parameters were set as follows: (1) formula error of 0.5 ppm, (2) relationship error of 20 ppm, and (3) mass limit of 500 Da. See S1 Table for elemental formula assignment information.

### Data Handling

All peaks found in the solvent blank were removed from controls and samples. For discussions that focus on oil-derived compounds, peaks common to the seawater controls and oil-added treatments were removed. Statistical analyses were performed using the Fathom toolbox [52] in MATLAB.

## Results and Discussion

### Overall Chemical Composition of WAF and WSF

**Fig 1:**
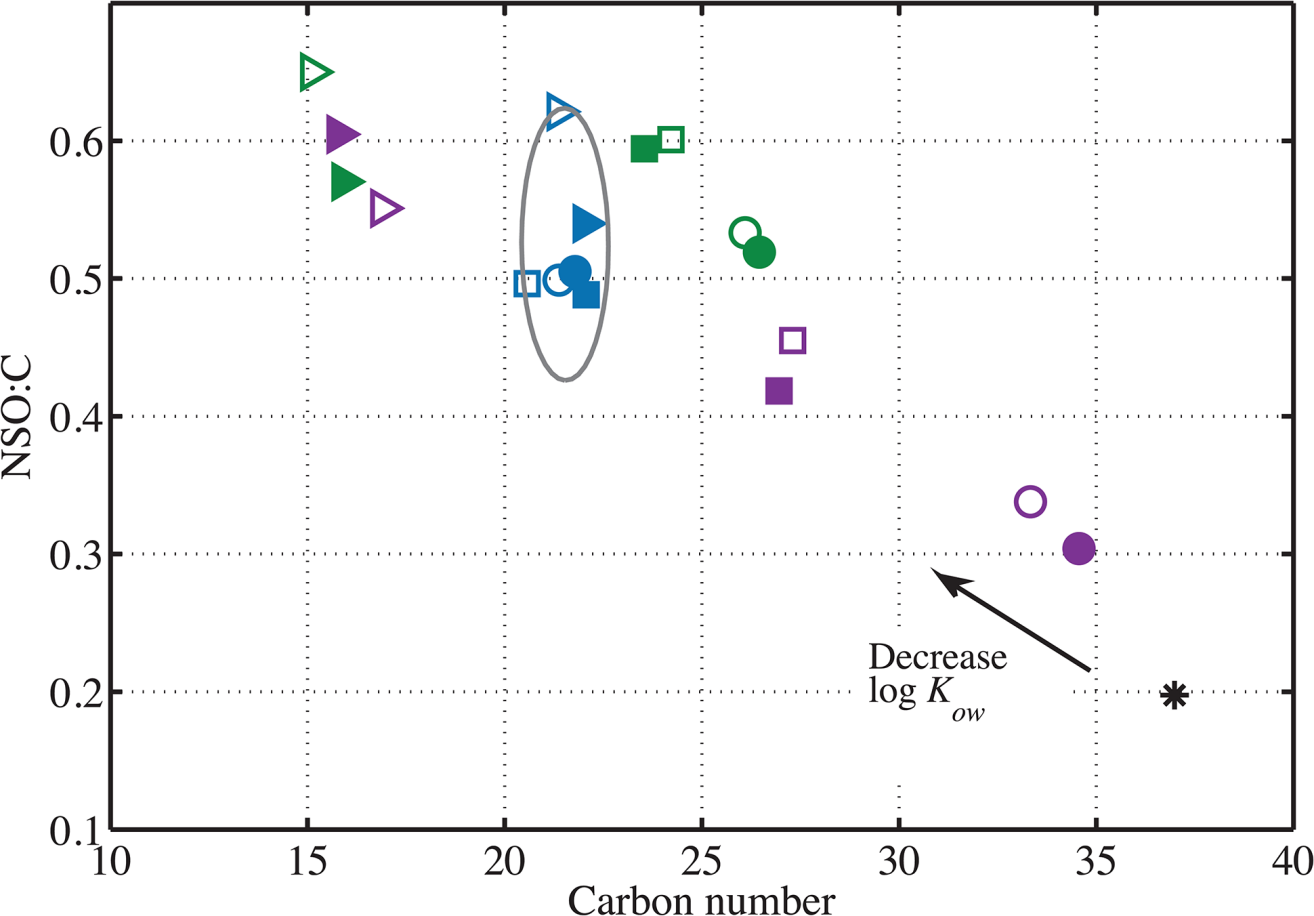
Number-averaged carbon number (C#) vs. NSO:C for each sample and control. Filled symbols indicate data collected from the VSW treatment and open symbols indicate data collected from the VSWE treatment. Circles, squares and triangles indicate DCM 1, DCM 2, and DCM-PPL extracts, respectively. Each extract of the seawater control is plotted in blue. The seawater controls are clustered together within a 95% confidence ellipse. Water-accommodated fractions (WAF) are plotted in purple symbols. Water-soluble fractions (WSF) are plotted in green symbols. The crude oil control is represented by a black asterisk.

**Fig 2:**
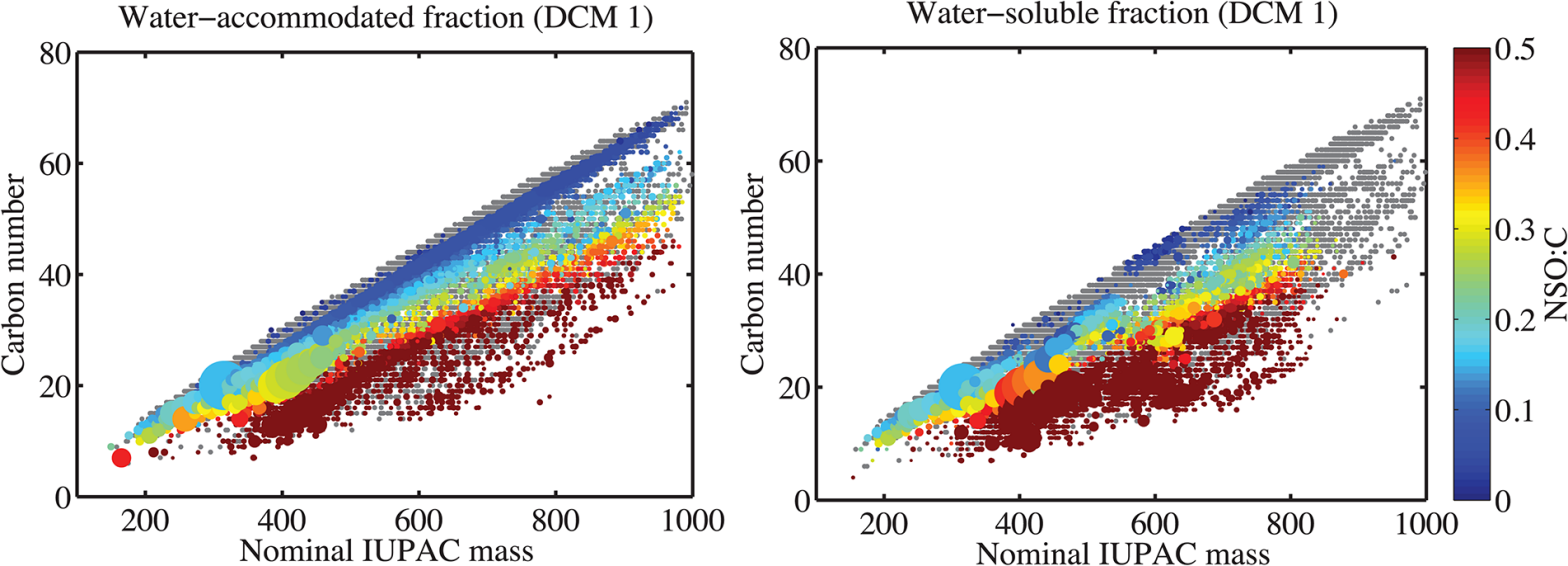
Carbon number vs. nominal mass plot of the DCM 1 extracts of the water-accommodated fraction (WAF; left) and water-soluble fraction (WSF; right) from the VSW treatment. Each *m/z* value with an assigned elemental formula is represented by a dot on the figure. The size of each dot corresponds to relative peak height. Components in parent oil are plotted in grey to serve as reference. Color bar indicates ratio of total heteroatoms to carbon atoms (NSO:C).

**Fig 3:**
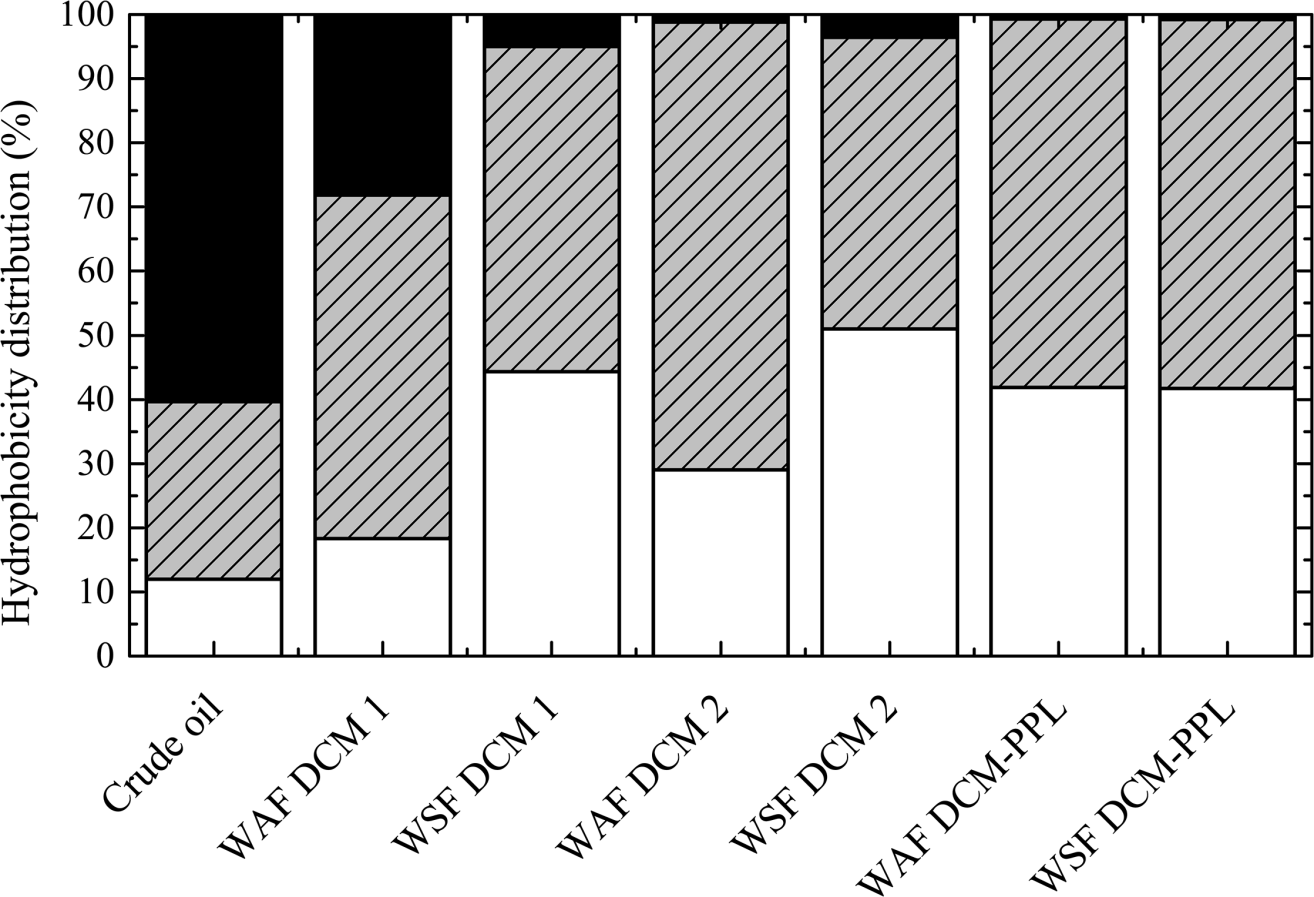
Distribution of hydrophilic (NSO:C ≥ 0.49 and molecular mass < 850 Da; white), moderately hydrophobic (0.1 < NSO:C < 0.49 and molecular mass < 850 Da; grey striped), and hydrophobic (NSO:C ≤ 0.1 or molecular mass > 850 Da; black) compounds in the crude oil control and the three extracts of the water-accommodated fraction (WAF) and the water-soluble fraction (WSF) from the VSW treatment. Hydrophobicity distribution is calculated as the percentage of total formulas from each hydrophobicity class relative to all formulas in a given sample.

The DCM 1, DCM 2 and DCM-PPL extracts of the aqueous samples (i.e. WAF and WSF) are compositionally different from the parent crude oil (Fig 1 and S1 Fig). Furthermore, WAF samples are compositionally distinct from their WSF counterparts within the DCM 1 and DCM 2 extracts (Figs 1, 2 and 3; S1 and S3 Figs). We conclude that filter-adsorption, therefore, had a significant impact on oil-derived lipophilic compound composition in the aqueous phase. In contrast, filtration did not noticeably affect the very polar fractions (i.e. DCM-PPL) of WSF due to their hydrophilic nature (Fig 1 and S1 Fig). Between the VSW and VSWE treatments, no significant compositional differences were observed between pairs of extracts (e.g. DCM 1’s), suggesting that lipophilic DOM constituents removed by DCM do not noticeably affect the solubility of crude oil-derived polar compounds (Fig 1; S1 Fig and S1 Table).

We used carbon number vs. nominal mass (CvM) plots [19] to visualize our data. At a given nominal mass in the CvM plots, a higher carbon number indicates lower hydrogen and/or heteroatom content [19]. Thus, the CvM plots enabled visualization of heteroatom contributions and aromaticity as a function of molecular size and thus depicted clear distinctions among our samples. The most prominent feature of the crude oil CvM plot is the presence of compounds with low heteroatom content (NSO:C < 0.1; S2 Fig). These compounds account for 56% of all detected compounds with elemental formulas assigned in the crude oil control, and are dominated by the N_1_, O_1_, O_2_, and N_1_O_1_ heteroatom classes. In contrast, the dominant heteroatom classes in all WAF and WSF extracts contain at least three heteroatoms per molecule (S2 Table).

Few compounds with NSO:C < 0.1 are observed in any aqueous extracts, except for WAF DCM 1 (Fig 2 and S3 Fig). Compounds with NSO:C < 0.1 account for 22% of all crude oil-derived compounds detected in WAF DCM 1 under ESI (-); in contrast, these compounds comprise less than 4% of all crude oil-derived compounds in all other aqueous extracts. Compounds with NSO:C < 0.1 in WAF DCM 1 overlap with the crude oil control (Fig 2 and S2 Fig). This unique feature marks the key distinction between WAF DCM 1 and its DCM 2 and WSF counterparts. In addition, a mass limit of ~850 Da was observed in WAF DCM 2 and all DCM extracts of WSF in this study as well as in our preliminary test with the West Texas oil (Fig 2 for DCM 1; S3 Fig for DCM 2 and S8 Fig for preliminary test).

Collectively, these findings imply that (1) the dominant compounds in crude oil do not effectively partition into the aqueous phase, which is consistent with results from Stanford et al. [11]; and (2) there is a lower NSO:C limit and an upper mass limit for aqueous partitioning of polar crude oil-derived compounds in our experiment. NSO:C serves as a proxy for the polarity of a given compound, because heteroatom-containing functional groups enhance polarity of organic compounds due to their high electronegativity relative to a carbon atom. Indeed, our results show that the most significant factors driving the differences between the samples and controls as well as among different extracts are the NSO:C value, a proxy for polarity, and carbon number, a proxy for solute size (Fig 1). Thus, we will focus the following sections on developing an assessment of chemical properties of dissolved and partially dissolved crude oil components, in contrast to the properties of crude oil and background seawater DOM. This approach offers a general model based on the principles of physical chemistry and thus can be applied to estimate the environmental impact of anthropogenic oil spills and natural oil seepage.

### Partitioning of Crude Oil-Derived Compounds into Seawater

Crude oils are complex mixtures with thousands of compounds [13] and their compositions differ across geographic sources and degrees of thermal maturity [12]. However, the water-soluble compounds derived from oils of different origins appeared to share similar characteristics such as heteroatom classes and mass range [11], suggesting their ability to partition into the aqueous phase is governed by common physical chemical properties.

We have developed a empirical model based on the octanol-water partition coefficients (log *K*_*ow*_) estimated from a series of compounds with known chemical properties and structures, including those found in crude oil [11, 12, 14], to guide our data interpretation (full details in S1 Text). Briefly, 214 heteroatom-containing model compounds were selected based on following criteria: (1) compounds known to exist in petroleum based on prior studies [14], (2) natural organic compounds with known solubility behavior, (3) possible functional groups combined with known core structures in petroleum, and (4) chemical structures that are possible in petroleum and yielded the same chemical formula as those detected in our data. Examples of chemical structures used in this study are shown in S4 Fig

Log *K*_*ow*_ describes the hydrophobicity of a given organic compound [53] and is related to water solubility (*S*) [54]. In general, log *K*_*ow*_ is affected by solute molecular size, polarity, and hydrogen bond strength of a given compound. We used the fragment contribution method [53, 55] to estimate log *K*_*ow*_ of the model compounds in this study. In this model, addition of aliphatic and aromatic carbon increases log *K*_*ow*_ (S5 Fig) and addition of heteroatom-containing functional groups generally decreases log *K*_*ow*_ (i.e. have a negative fragment contribution coefficient; S3 Table). A higher log *K*_*ow*_ value indicates higher hydrophobicity and hence lower aqueous solubility.

Solute size has a significant impact on log *K*_*ow*_. There are significant correlations between log *K*_*ow*_ and molecular mass or carbon number for hydrocarbons containing only carbon and hydrogen (S5 Fig). For pure *n*-alkanes, aqueous solubility is exceedingly small and can be considered negligible for compounds with more than 16 carbon atoms [32]. Aromatic hydrocarbons are in general more water-soluble than aliphatic hydrocarbons; for example, a C_16_ aromatic hydrocarbon such as pyrene (molecular mass 202.25 Da) is more water-soluble (sparingly soluble) than its insoluble *n*-alkane counterpart (S5 Fig). Nonetheless, given the sparing solubility of pyrene, its dissolved concentrations are usually very low [56], unless it is solubilized by other organic compounds in the aqueous phase [43]. In contrast to the solute size considerations, most of the compounds detected in the aqueous phase with our analytical method have a molecular mass higher than that of pyrene and thus we conclude that their presence in WAF and WSF cannot be due solely to enhanced aromaticity.

**Fig 4:**
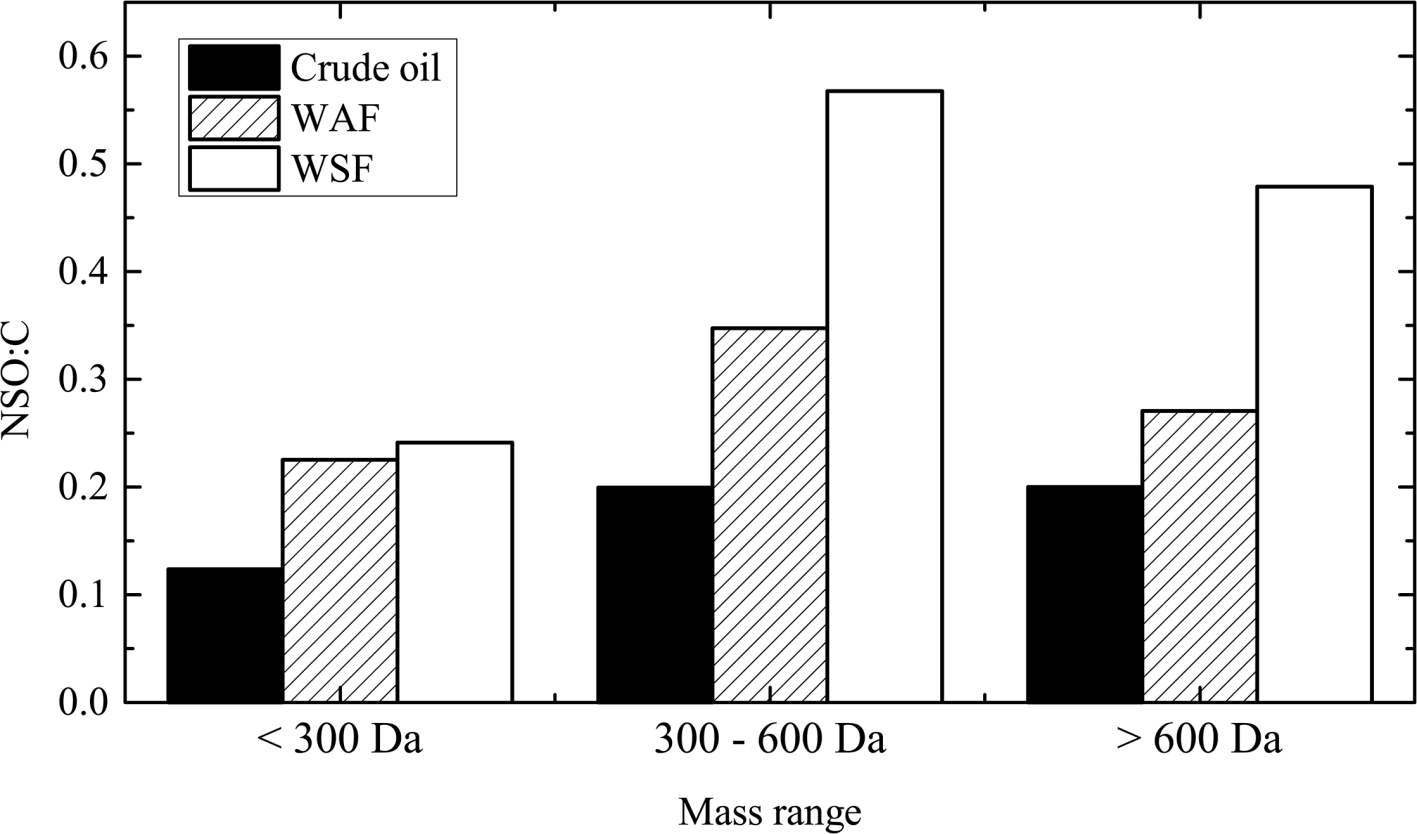
Number-averaged NSO:C values of crude oil (black) and the DCM 1 extracts of the water-accommodated fraction (WAF; striped) and the water-soluble fraction (WSF; white) from the VSW treatment in the mass ranges: < 300 Da, 300 to 600 Da and > 600 Da.

Instead, the heteroatom content in the polar compounds likely plays a key role in their aqueous partitioning. The log *K*_*ow*_ values estimated with the model compounds exhibit a significant negative correlation with NSO:C (Pearson’s *r* = -0.81; *p* < 0.001), within the heteroatom content range considered in this study (S6 Fig). Based on the current knowledge of possible chemical structures of crude oil-derived compounds [11–14], it would be reasonable to assume that in most cases higher heteroatom content offsets the solute size effect and hence favors the chemical partitioning of these compounds into the aqueous phase. Indeed, the number-averaged NSO:C across our detected molecular mass range showed that DCM 1 extracts of the aqueous fractions have higher NSO:C values than the crude oil control (Fig 4). At molecular mass > 300, NSO:C values in WSF are significantly higher than in WAF. These findings confirmed the effect of heteroatom content in aqueous partitioning, indicating that higher NSO:C is required for high molecular weight compounds to remain dissolved or partially dissolved in seawater. The same trend was observed in DCM 2 (S7A Fig). In contrast, NSO:C is not significantly different between DCM-PPL samples across the mass range (S7B Fig).

### Shift in Solute Size and NSO:C across Extracts

We found that the bulk compositional properties of our samples differ along the gradients of carbon number (C#) and NSO:C (Fig 1). Lower C# and elevated NSO:C would likely decrease log *K*_*ow*_ and hence favor enhanced aqueous partitioning of the crude oil-derived compounds. All aqueous samples and seawater controls have higher number-averaged NSO:C values and lower C# than the crude oil control (Fig 1). This implies that low molecular weight polar crude oil compounds were selectively partitioned into the aqueous phase. Sequential extractions and filter adsorption further shift the WAF DCM 2 and WSF samples, respectively, toward lower C# and higher NSO:C (Fig 1), suggesting these treatments effectively remove relatively hydrophobic, or more lipophilic, compounds.

Molecular level information revealed that WAF DCM 1 contains significantly more hydrophobic compounds (NSO:C ≤ 0.1 or molecular mass > 850 Da; see discussion below), and thus is more similar to the crude oil, than DCM 2 and its WSF counterparts (Fig 2; S3 Fig and S1 Table). Therefore, the C# and NSO:C shift of WAF DCM 1 relative to crude oil is not as drastic compared to the other aqueous samples. Compounds with low NSO:C and/or molecular mass > 850 Da were readily removed by solvent extraction and filter adsorption and likely represent compounds that are present only in microscopic oil droplets in WAF.

In the DCM-PPL, most compounds are less than ~450 Da (S3 Fig). After the two sequential solvent extractions, the smaller and more polar compounds are enriched in the DCM-PPL fraction, relative to DCM 1 and 2 (Fig 1 and S3 Fig). It should be noted that the sequential solvent and solid phase extractions did not result in large changes in C# and NSO:C among seawater control extracts, suggesting seawater DOM may have less distinct NSO:C and C# signatures across the different extracts. Sequential extractions lead to different NSO:C and C# patterns between dissolved crude oil compounds and marine DOM, suggesting that these two pools of organic compounds possess different chemical properties.

### Hydrophobicity and Potential Environmental Impacts

We divided our compositional data into three classes: (1) hydrophobic compounds (NSO:C ≤ 0.1 or molecular mass > 850 Da), (2) moderately hydrophobic compounds (0.1 < NSO:C < 0.49 and molecular mass < 850 Da), and (3) hydrophilic compounds (NSO:C ≥ 0.49 and molecular mass < 850 Da). The hydrophobic class defined above includes compounds that are likely insoluble or sparingly soluble and represents compounds that were removed by solvent extraction and filter adsorption. Based on an empirical relationship between log *K*_*ow*_ and NSO:C (S1 Text and S6 Fig), compounds with NSO:C = 0.1 corresponded to a modeled log *K*_*ow*_ value of 4.86 ± 0.27. This range of log *K*_*ow*_ is similar to that of pyrene, a sparingly soluble hydrocarbon in seawater (measured log *K*_*ow*_ = 4.88; calculated log *K*_*ow*_ = 4.93) [57], which validates our NSO:C criterion for the hydrophobic class. The heteroatom criterion for the hydrophilic class was based on the lowest number-averaged NSO:C value of the seawater controls (Fig 1), assuming all organic constituents in seawater are predominantly dissolved.

Although the polar petroleum components should be relatively more water-soluble than the non-polar fraction, our results showed that the polar fraction of crude oil contains a substantial number of compounds that are likely insoluble or sparingly soluble (Fig 3). Since polar compounds account for < 15% of crude oil by mass [12], we conclude that only a small mass fraction of crude oil is likely soluble in seawater. Nonetheless, this small proportion of crude oil is chemically diverse and could have significant and prolonged impacts on the seawater DOM pool. A recent study reported that water impacted by the DWH oil spill showed higher dissolved organic carbon (DOC) concentrations than the typical DOC background observed in the Gulf of Mexico, two years after the oil spill [58]. This pool of recalcitrant petrogenic DOM observed in the Gulf of Mexico could be polar compounds from the original or weathered oil, as they are known to be more resistant to different degradation processes [5].

In the aqueous phase, WAF DCM 1 has the highest proportion of hydrophobic compounds due to the presence of low NSO:C compounds. These compounds may present in dispersed microscope oil droplets or partially dissolved in the seawater, which is relevant to marine or aquatic oil spills [5]. These hydrophobic compounds were readily removed by solvent extraction, which suggests that at least a portion of them may partition into organic phases such as bio-fluids, lipid membranes, or particulate organic matter. Filter adsorption also removed most hydrophobic compounds, suggesting the potential to adsorb to inorganic particles.

Due to their poor water-solubility and the potential to interact with particles, the toxicological impacts of highly hydrophobic compounds to marine organisms may depend on the time-scale. It has been shown that bioconcentration factor (BCF) decreases for highly hydrophobic compounds, while BCF is positively correlated with hydrophobicity for hydrophilic and moderately hydrophobic compounds [59]. This effect is presumably due to limited bioavailability as highly hydrophobic compounds are often insoluble. In fact, a prior study showed that dissolved oil components are more toxic to fish embryos than oil droplets [42]. Thus, we postulate that the hydrophobic compounds, which comprise a significant fraction of WAF, have limited short-term toxicological impacts to marine organisms such as macrofauna in the water column. However, hydrophobic compounds may preferentially partition into, accumulate, and potentially persist in organic sediments [60]. Therefore, these hydrophobic compounds may have long-term effects in marine ecosystems.

In contrast to WAF DCM 1, WAF DCM 2 and the other aqueous extracts contain a larger fraction of moderately hydrophobic and hydrophilic compounds (Fig 3). Due to the relatively lower hydrophobicity, compounds in these classes are expected to be completely dissolved and hence more bioavailable. The moderately hydrophobic compounds are dissolved in the aqueous phase but have a high tendency to partition into the organic phase. Compounds with moderate hydrophobicity may pose a greater immediate threat to macrofauna than the highly hydrophobic or hydrophilic compounds as they are soluble enough to stay in water and their hydrophobicity facilitates partitioning into lipid membranes of an animal [61]. On the other hand, the hydrophilic compounds may pose the least threat to macrofauna as they are very soluble and hence do not readily partition into organic phases like the membranes of animals. Because of their higher probability to stay in the aqueous phase, hydrophilic compounds are more likely to encounter bacteria and be degraded or biotransformed. The hydrophilic compounds in the DCM-PPL fraction may be particularly important with regards to microbial interactions. DCM-PPL contained primarily low molecular weight compounds (208–800 Da; median 360 Da). The majority of these compounds have a molecular mass between 300 and 400 Da, which is small enough to be directly assimilated by microorganisms [62].

WAF and WSF are often used interchangeably in toxicology studies. Although WAF is the fraction that most closely resembles oil spill scenarios, it is essential to distinguish WSF from WAF in order to isolate the fraction containing toxic constituents for further study. Thus, elucidation of the molecular-level differences between WAF and WSF, as shown in this study, is a significant contribution to the environmental scientific community. Our results highlight the importance of a consistent preparation method for creating the dissolved fraction for toxicological and chemical studies that assess oil-derived compounds in aquatic systems. Because WAF and WSF contain different proportions of compounds from our three general hydrophobicity classes, it is reasonable to expect that they will have distinct toxicological impacts on exposed organisms.

### Solubilization Effect of Organic Compounds in Dissolved Phase

A small portion of hydrophobic compounds was observed in the WSF samples (Figs 2 and 3). These compounds, which have relatively high molecular mass and low heteroatom content, are practically insoluble in seawater. The presence of insoluble compounds in WSF may be partially attributed to incomplete removal of partially dissolved compounds with the GF/F filter. However, it is more likely due to solubilization by dissolved crude oil components with surfactant properties (e.g. compounds containing sulfonic groups) [11] or by marine DOM (e.g. humic and fulvic acids and organic colloidal materials) [30].

The latter hypothesis is supported by numerous prior studies. Humic and fulvic substances are known to solubilize insoluble contaminants in the environment [63, 64] by incorporating these organic compounds into hydrophobic spaces within the macromolecular structures [65]. Boehm and Quinn found that removing natural DOM in seawater reduced dissolved *n*-alkane and isoprenoid hydrocarbons by 50 to 99% [30]. Solubilization tests showed that addition of humic and fulvic acids increased the amounts of sparingly soluble (e.g. pyrene) [43] and insoluble (e.g. pristane) [30] hydrocarbons dissolved in seawater. In contrast, pyrene concentrations were negligible without humic acid addition [43]. A recent study showed that colloidal materials with amphiphilic properties, such as extracellular polymeric substances (EPS), are also able to solubilize hydrocarbons in seawater [66].

The seawater used in this study was collected from the coast, where humic and fulvic acids and colloidal materials are expected to present at considerable concentrations. Therefore, the small proportion of insoluble compounds observed in WSF and in the more hydrophilic fractions (i.e. DCM-PPL) may be explained by solubilization by certain classes of seawater DOM. Lipids should enhance solubility of the hydrophobic compounds. However, lipid-removal in the VSWE treatment did not affect the chemical compositions of WAF and WSF. These findings suggest that the relatively low abundance of DCM-extractable lipids in natural seawater does not have a significant impact on oil component solubility.

The solubilization effect of DOM will have significant toxicity implications as it increases the apparent aqueous solubility of hydrophobic compounds. An experiment conducted by Boehm and Quinn demonstrated that DOM solubilization of petroleum hydrocarbons decreased the amount of insoluble compounds retained on a glass fiber filter and the amount of hydrocarbons assimilated by a bivalve [31]. Although the incorporation of petroleum hydrocarbons into humic acids has equivocal effects on subsequent microbial degradation [67, 68], it has been shown that humic acids could enhance biodegradation of polycyclic aromatic hydrocarbons in the aqueous phase [67]. Organic colloidal materials, e.g. amphiphilic EPS, have also been shown to enhance biodegradation of hydrocarbons in seawater [66] and may be more important in open-ocean settings where humic and fulvic acid concentrations are low. Thus, interactions with DOM may play an important role in regulating crude oil-derived compounds’ toxicity to marine organisms and degradability by bacteria.

### Environmental Implications of Polar Crude Oil Components and Future Considerations

Our results from the oil dissolution experiment presented the first assessment of aqueous solubility and potential environmental impacts of polar crude oil compounds based on state-of-the-art FT-ICR-MS molecular level analysis. Our results elucidated multiple processes that can affect environmental impacts of polar crude oil components. In an oil spill, the compositional distribution of polar compounds will likely be affected by the spilled oil’s geographical origin and degree of maturity. More importantly, the hydrophobicity class distributions, which are expected to affect toxicity of the oil to marine organisms, will likely differ among various oil sources and oil spills. The relative proportion of different hydrophobicity classes is controlled by physical/chemical properties of the oil as well as DOM in seawater.

As discussed above, humic and fulvic acids and some amphiphilic organic colloidal materials are important DOM constituents in regulating solubility of crude oil-derived compounds, toxicity to macro- and microorganisms, as well as biodegradability of these compounds. Thus, the quality, quantity, and environmental impacts of polar crude oil components may depend on oil spill location. For example, if an oil spill occurred near shore, where humic and fulvic acid supplies are ample, the amounts and diversity of dissolved hydrocarbons may increase in comparison to offshore oil spills. The solubilization effect of seawater DOM may substantially alter the polar crude oil components’ apparent toxicity and bioavailability to marine organisms. Therefore, interactions between DOM and polar crude oil components should be further investigated.

Our results show that crude oil-derived polar compounds are compositionally distinct between the WAF and WSF. It is, therefore, crucial to be cautious when deciding which fraction to use in a given study. The choice of using WAF vs. WSF should be based on the study objectives. For example, it is important to consider WAF with different oil droplet sizes and quantities if the goal is to mimic realistic oil spill scenarios. On the other hand, it is essential to distinguish between WAF and WSF when considering chemical characterization, microbial degradation and responses, and organismal toxicity of crude oil components.

Finally, weathering processes are known to enhance heteroatom content of crude oil components (i.e. formation of oxygenated compounds) and hence should alter their hydrophobicity. Whether the degradation processes yield more or less toxic compounds with different environmental residence times would depend on their resulting structure and thus parameters that govern hydrophobicity (i.e. log *K*_*ow*_). Moreover, crude oils contain thousands of compounds with diverse carbon number distributions. Thus, addition of oxygen during weathering alters each compound’s hydrophobicity and toxicity in a potentially different way. Both compositional and structural information on polar crude oil components and their weathering products are required for better assessment of environmental impact of oil spills.

## Supporting Information

S1 Fig Non-metric multidimensional scaling analysis of the samples and controls examined in this study, based on all detected masses in each sample. (PDF)

S2 Fig Carbon number vs. nominal mass plot of crude oil (left) and the DCM 1 extract of the VSW seawater control (right). Each *m/z* value with an assigned elemental formula is represented by a dot on the figure. The size of each dot corresponds to relative peak height. Components in parent oil are plotted in grey as reference. Color bar indicates ratio of total heteroatoms to carbon atoms (NSO:C).
(PDF)

S3 Fig Carbon number vs. nominal mass plot of DCM 2 (upper) and DCM-PPL (lower) extracts of water-accommodated fraction (WAF; unfiltered; left) and water-soluble fraction (WSF; filtered; right) from the VSW treatment. Each *m/z* value with an assigned elemental formula is represented by a dot on the figure. The size of each dot corresponds to relative peak height. Components in parent oil are plotted in grey as reference. Color bar indicates ratio of total heteroatoms to carbon atoms (NSO:C).
(PDF)

S4 Fig Examples of chemical structures used in the log *K*_*ow*_ estimation: (1) known structures in petroleum presented by McKenna et al. (upper), (2) estimated structures based on known petroleum structures and assigned formulas in our data (middle), and (3) naturally occurring heteroatom-containing compounds: riboflavin, tryptophan and folate (lower). (PDF)

S5 Fig Molecular mass vs. octanol-water partition coefficient (log *K*_*ow*_) for C_6_ to C_20_ n-alkanes (grey: hexane, decane, tetradecane, hexadecane, and eicosane) and aromatic hydrocarbons (black: benzene, naphthalene, phenanthrene, pyrene, and benzo[a]pyrene). (PDF)

S6 Fig Scatter plot of NSO:C vs. log *K*_*ow*_ estimated from a series of model structures. Black filled circles represent structures shown in S4 Fig Red line indicates the linear fit of the data; solid grey lines are the upper and lower 95% confidence limits; and dotted grey lines are the 95% prediction interval, which estimates the range of future observations.
(PDF)

S7 Fig Number-averaged NSO:C values of DCM 2 (a) and DCM-PPL (b) extracts of the water-accommodated fraction (striped) and the water-soluble fraction (white) from the VSW treatment in the mass ranges: < 300 Da, 300 to 600 Da, and > 600 Da. (PDF)

S8 Fig Carbon number vs. nominal mass plot of West Texas crude oil (upper left), Vineyard Sound seawater DCM 1 (upper right; collected in summer 2010), and water-accommodated fraction generated from West Texas oil (lower left). Each *m/z* value with an assigned elemental formula is represented by a dot on the figure. The size of each dot corresponds to relative peak height. Components in parent oil are plotted in grey as reference. Color bar indicates ratio of total heteroatoms to carbon atoms (NSO:C).
(PDF)

S1 Table Bulk chemical features of samples and controls. Hydrophobicity classes are define as follows: hydrophilic compounds (NSO:C ≥ 0.49 and molecular mass < 850 Da), moderately hydrophobic compounds (0.1 < NSO:C < 0.49 and molecular mass < 850 Da), and hydrophobic compounds (NSO:C ≤ 0.1 or molecular mass > 850 Da).
(PDF)

S2 Table Heteroatom classes with relative abundance > 1% in the seawater-oil mixed samples. (PDF)

S3 Table Selected fragment contribution coefficients for functional groups likely present in crude oil and petroleum-derived products. The coefficient values are obtained from the EPA EPI Suite.
(PDF)

S1 Text Log *K*_*ow*_ estimation method. (PDF)
