# Supplementary material for: Chemical Composition and Potential Environmental Impacts of Water-Soluble Polar Crude Oil Components Inferred from ESI FT-ICR MS
Source: PLoS One. 2015 Sep 1;10(9):e0136376. doi: 10.1371/journal.pone.0136376 (PMC4556654; doi:10.1371/journal.pone.0136376)
Supplement: S1 Fig — (PDF) [file pone.0136376.s001.pdf]

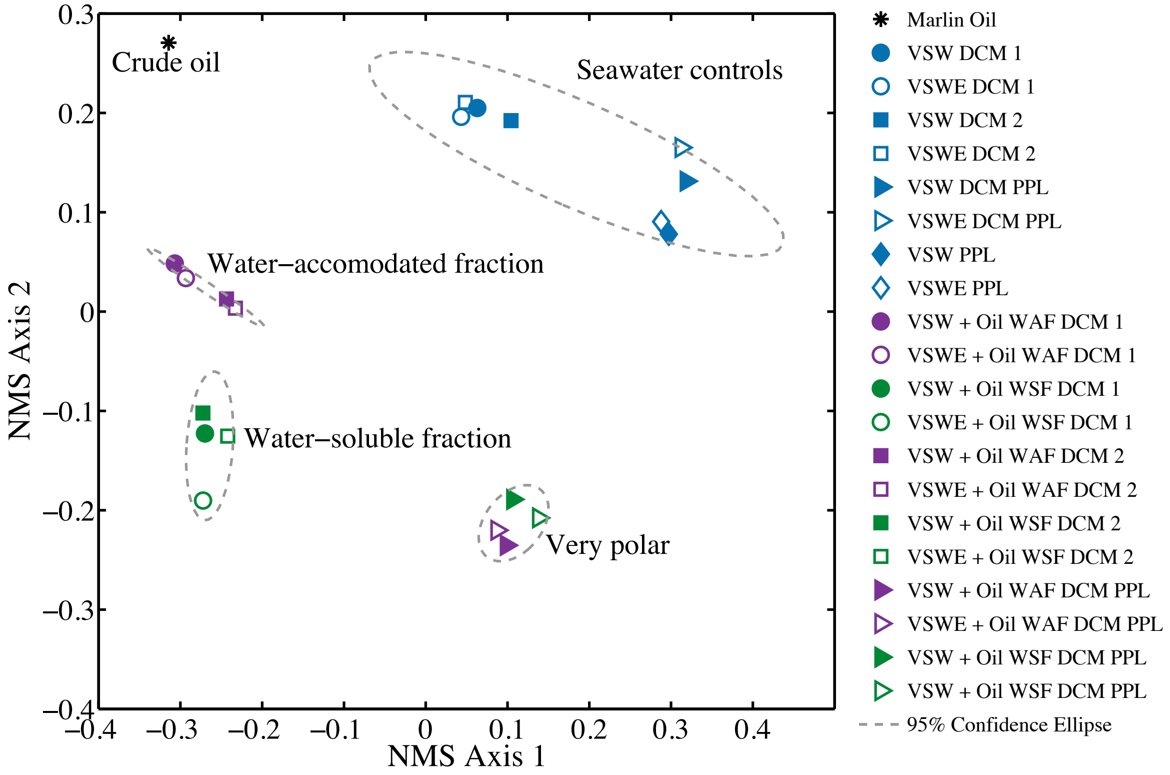

S1 Fig. Non-metric multidimensional scaling analysis of the samples and controls examined in this study, based on all detected masses in each sample.
