# Supplementary material for: Chemical Composition and Potential Environmental Impacts of Water-Soluble Polar Crude Oil Components Inferred from ESI FT-ICR MS
Source: PLoS One. 2015 Sep 1;10(9):e0136376. doi: 10.1371/journal.pone.0136376 (PMC4556654; doi:10.1371/journal.pone.0136376)
Supplement: S3 Fig — Each m/z value with an assigned elemental formula is represented by a dot on the figure. The size of each dot corresponds to relative peak height. Components in parent oil are plotted in grey as reference. Color bar indicates ratio of total heteroatoms to carbon atoms (NSO:C). (PDF) [file pone.0136376.s003.pdf]

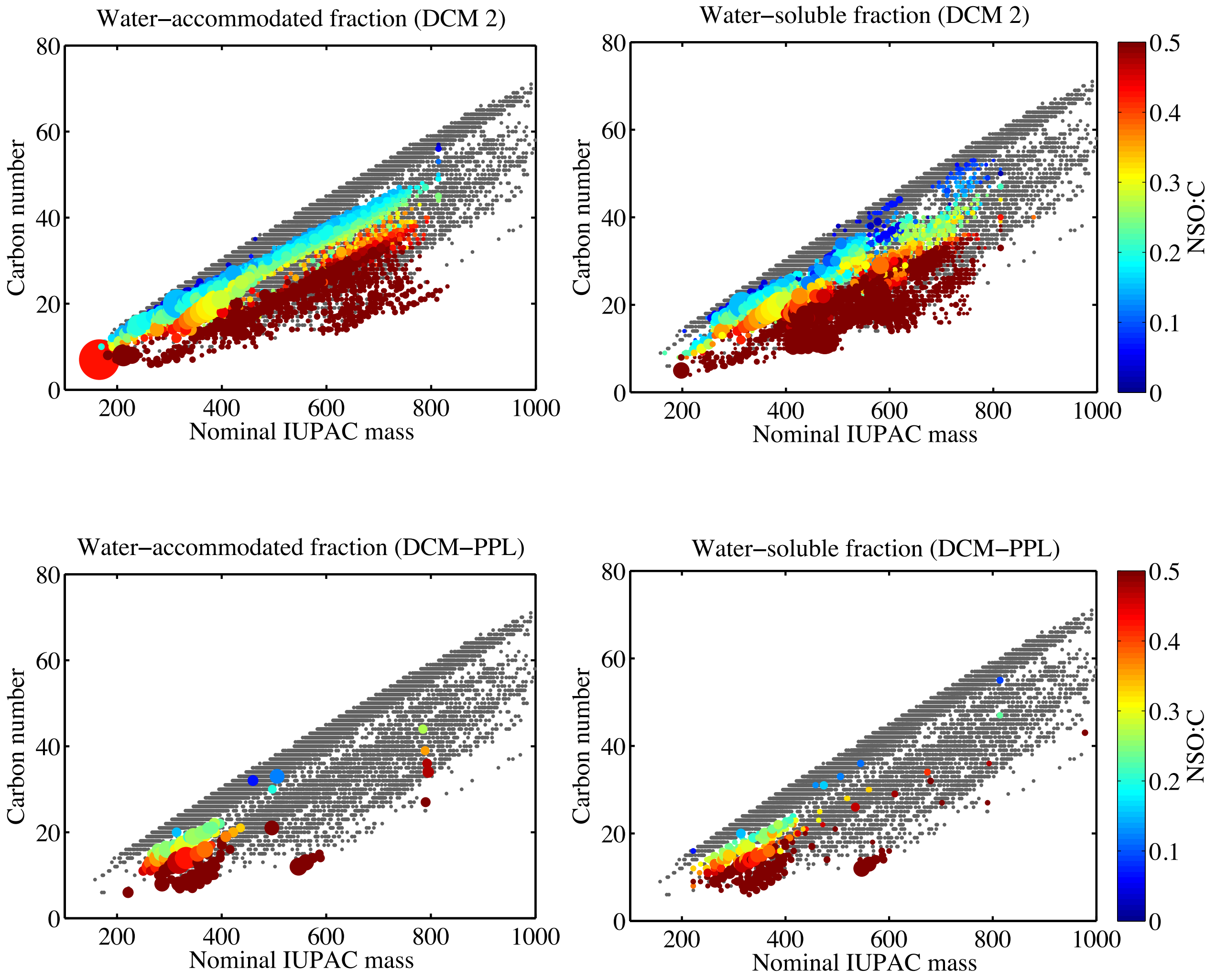

S3 Fig. Carbon number vs. nominal mass plot of DCM 2 (upper) and DCM-PPL (lower) extracts of water-accommodated fraction (WAF; unfiltered; left) and water-soluble fraction (WSF; filtered; right) from the VSW treatment. Each *m/z* value with an assigned

elemental formula is represented by a dot on the figure. The size of each dot corresponds to relative peak height. Components in parent oil are plotted in grey as reference. Color bar indicates ratio of total heteroatoms to carbon atoms (NSO:C).
