# Supplementary material for: Chemical Composition and Potential Environmental Impacts of Water-Soluble Polar Crude Oil Components Inferred from ESI FT-ICR MS
Source: PLoS One. 2015 Sep 1;10(9):e0136376. doi: 10.1371/journal.pone.0136376 (PMC4556654; doi:10.1371/journal.pone.0136376)
Supplement: S4 Fig — (PDF) [file pone.0136376.s004.pdf]

Examples of known structures in petroleum from McKenna et al., (2013)

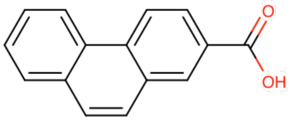

NSO:C = 0.13
log K_{ow} = 4.23

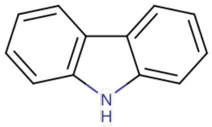

NSO:C = 0.08
log K_{ow} = 3.29

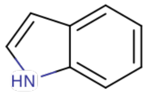

NSO:C = 0.13
log K_{ow} = 2.14

Examples of estimated structures based on known core structures and assigned formulas

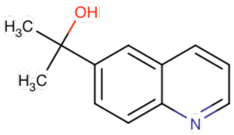

NSO:C = 0.17
log K_{ow} = 2.1

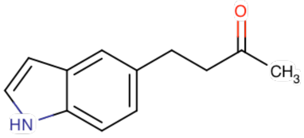

NSO:C = 0.17
log K_{ow} = 2.03

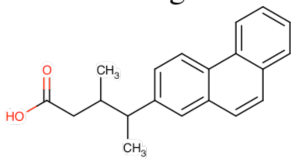

NSO:C = 0.10
log K_{ow} = 5.96

Examples of naturally occurring compounds used in the model

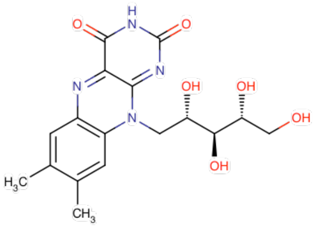

NSO:C = 0.59
log K_{ow} = -0.11

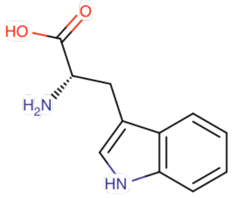

NSO:C = 0.36
log K_{ow} = -1.06

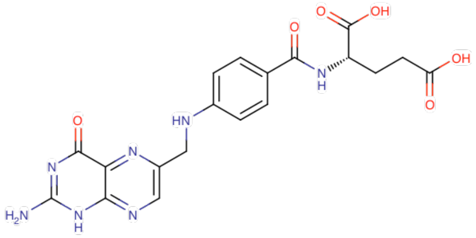

NSO:C = 0.68
log K_{ow} = -2.81

S4 Fig. Examples of chemical structures used in the log K_{ow} estimation: (1) known structures in petroleum presented by McKenna et al. [3] (upper), (2) estimated structures based on known petroleum structures and assigned formulas in our data (middle), and (3) naturally occurring heteroatom-containing compounds: riboflavin, tryptophan and folate (lower).
