# Supplementary material for: Chemical Composition and Potential Environmental Impacts of Water-Soluble Polar Crude Oil Components Inferred from ESI FT-ICR MS
Source: PLoS One. 2015 Sep 1;10(9):e0136376. doi: 10.1371/journal.pone.0136376 (PMC4556654; doi:10.1371/journal.pone.0136376)
Supplement: S5 Fig — (PDF) [file pone.0136376.s005.pdf]

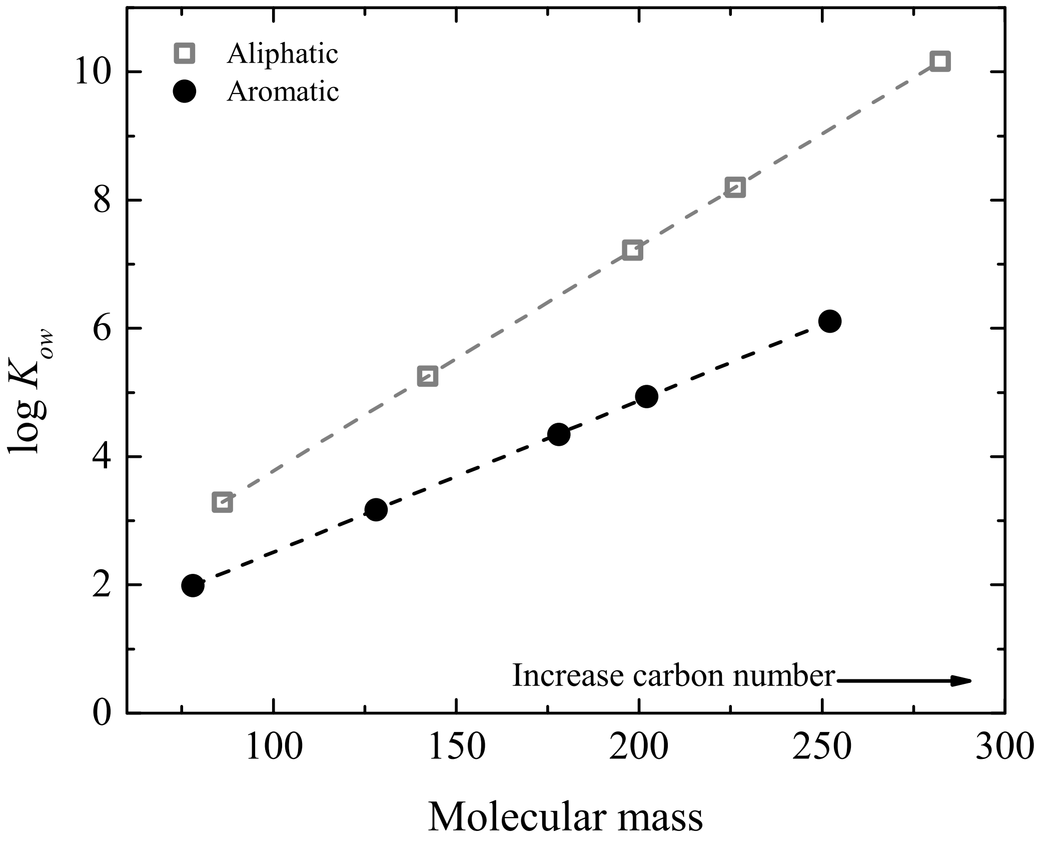

S5 Fig. Molecular mass vs. octanol-water partition coefficient (log K_{ow}) for C_6 to C_{20} n-alkanes (grey: hexane, decane, tetradecane, hexadecane, and eicosane) and aromatic hydrocarbons (black: benzene, naphthalene, phenanthrene, pyrene, and benzo[a]pyrene).
