# Supplementary material for: Chemical Composition and Potential Environmental Impacts of Water-Soluble Polar Crude Oil Components Inferred from ESI FT-ICR MS
Source: PLoS One. 2015 Sep 1;10(9):e0136376. doi: 10.1371/journal.pone.0136376 (PMC4556654; doi:10.1371/journal.pone.0136376)
Supplement: S6 Fig — Black filled circles represent structures shown in S4 Fig Red line indicates the linear fit of the data; solid grey lines are the upper and lower 95% confidence limits; and dotted grey lines are the 95% prediction interval, which estimates the range of future observations. (PDF) [file pone.0136376.s006.pdf]

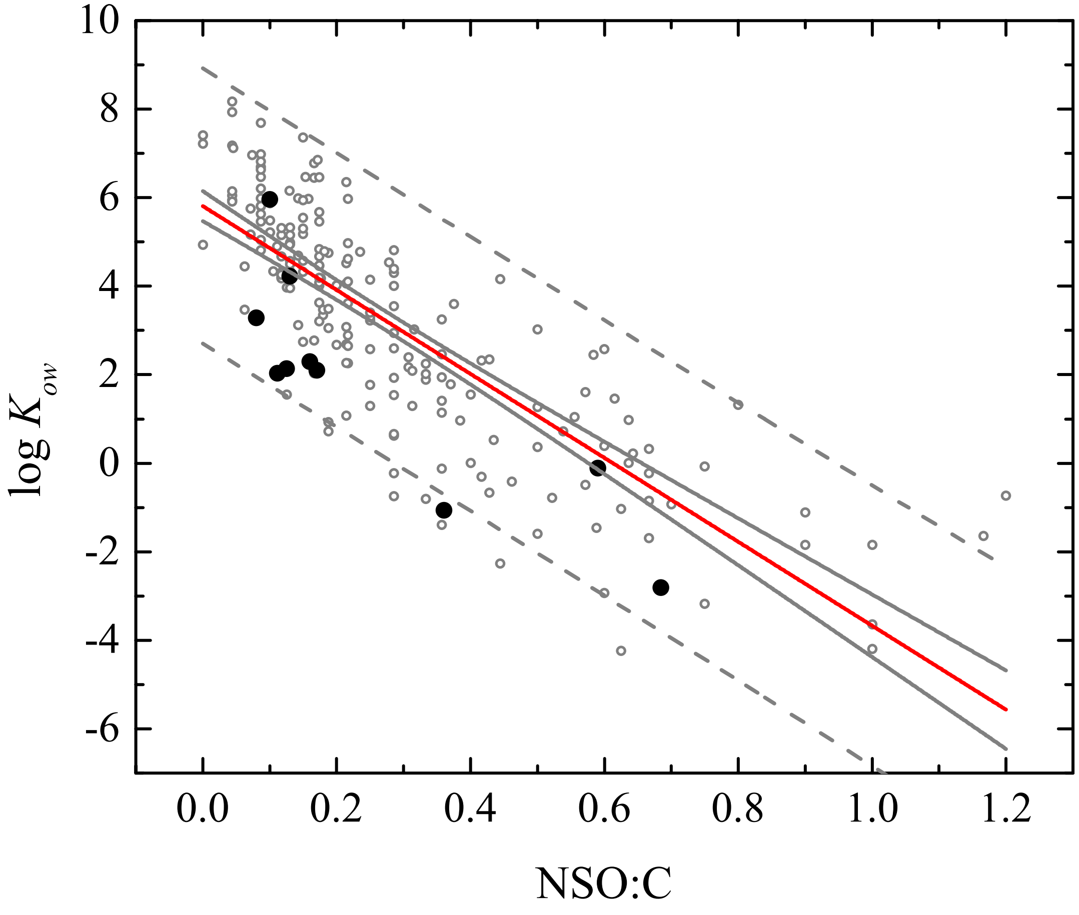

S6 Fig. Scatter plot of NSO:C vs. log K_{ow} estimated from a series of model structures. Black filled circles represent structures shown in S4 Fig. Red line indicates the linear fit of the data; solid grey lines are the upper and lower 95% confidence limits; and dotted grey lines are the 95% prediction interval, which estimates the range of future observations.
