# Supplementary material for: Chemical Composition and Potential Environmental Impacts of Water-Soluble Polar Crude Oil Components Inferred from ESI FT-ICR MS
Source: PLoS One. 2015 Sep 1;10(9):e0136376. doi: 10.1371/journal.pone.0136376 (PMC4556654; doi:10.1371/journal.pone.0136376)
Supplement: S7 Fig — (PDF) [file pone.0136376.s007.pdf]

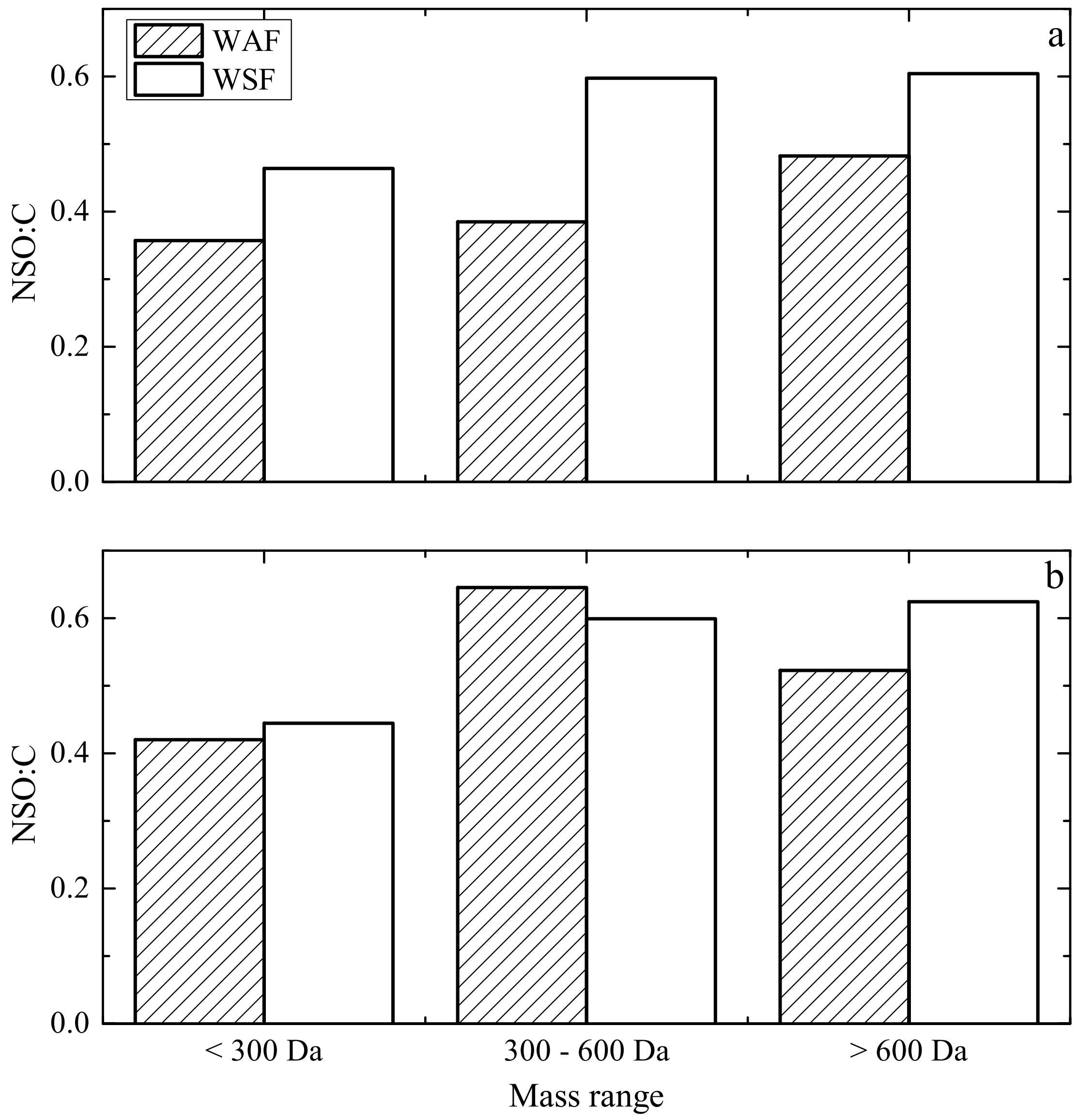

S7 Fig. Number-averaged NSO:C values of DCM 2 (a) and DCM-PPL (b) extracts of the water-accommodated fraction (striped) and the water-soluble fraction (white) from the VSW treatment in the mass ranges: < 300 Da, 300 to 600 Da, and > 600 Da.
