# Supplementary material for: Chemical Composition and Potential Environmental Impacts of Water-Soluble Polar Crude Oil Components Inferred from ESI FT-ICR MS
Source: PLoS One. 2015 Sep 1;10(9):e0136376. doi: 10.1371/journal.pone.0136376 (PMC4556654; doi:10.1371/journal.pone.0136376)
Supplement: S8 Fig — Each m/z value with an assigned elemental formula is represented by a dot on the figure. The size of each dot corresponds to relative peak height. Components in parent oil are plotted in grey as reference. Color bar indicates ratio of total heteroatoms to carbon atoms (NSO:C). (PDF) [file pone.0136376.s008.pdf]

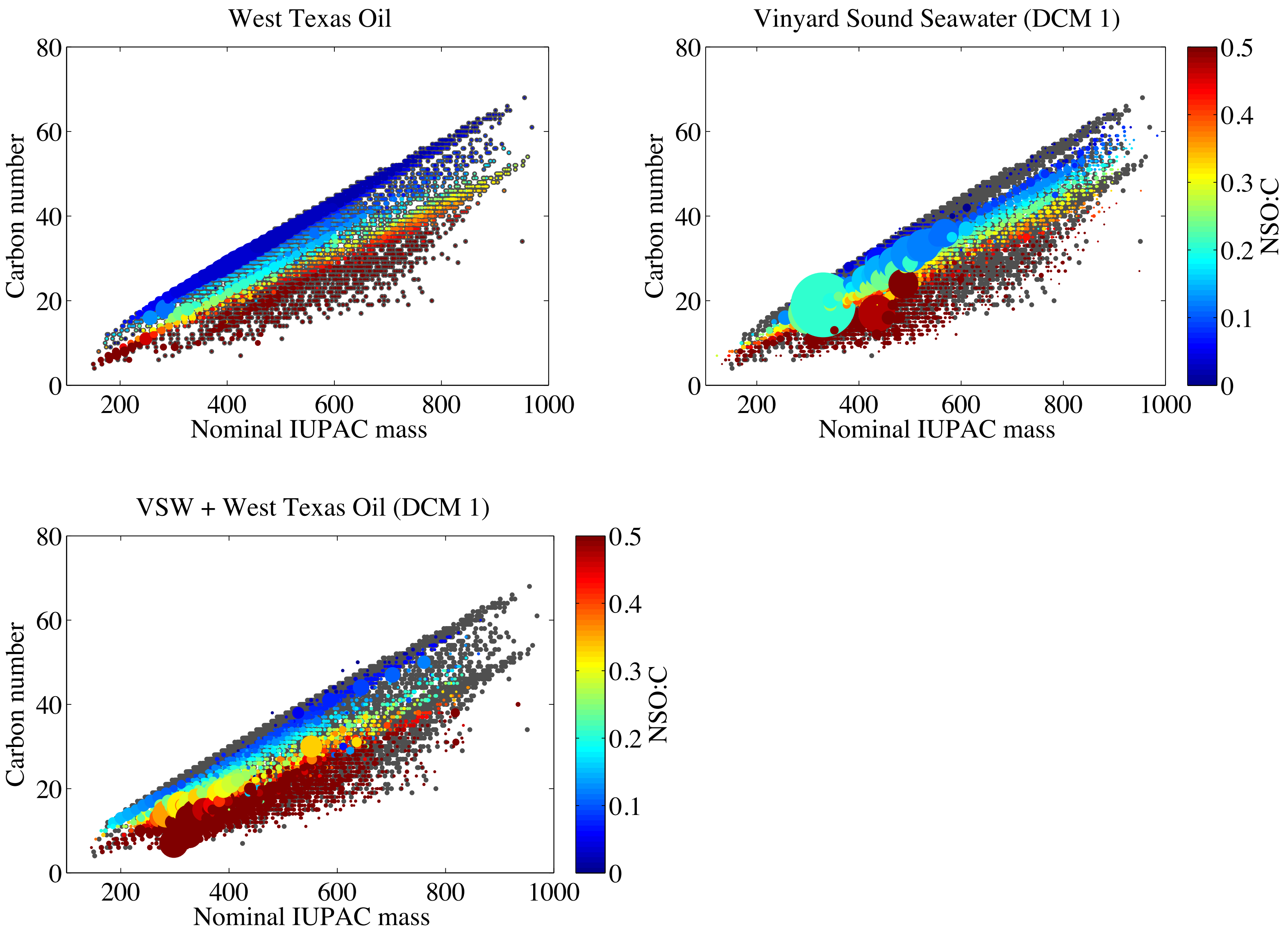
West Texas Oil
Vinyard Sound Seawater (DCM 1)
VSW + West Texas Oil (DCM 1)
Carbon number
Nominal IUPAC mass
NSO:C
0
20
40
60
80
200
400
600
800
1000
0.1
0.2
0.3
0.4
0.5

S8 Fig. Carbon number vs. nominal mass plot of West Texas crude oil (upper left), Vineyard Sound seawater DCM 1 (upper right; collected in summer 2010), and water-accommodated fraction generated from West Texas oil (lower left). Each *m*/*z* value with an assigned elemental formula is represented by a dot on the figure. The size of each dot corresponds to relative peak height. Components in parent oil are plotted in grey as reference. Color bar indicates ratio of total heteroatoms to carbon atoms (NSO:C).
