# Supplementary material for: Chemical Composition and Potential Environmental Impacts of Water-Soluble Polar Crude Oil Components Inferred from ESI FT-ICR MS
Source: PLoS One. 2015 Sep 1;10(9):e0136376. doi: 10.1371/journal.pone.0136376 (PMC4556654; doi:10.1371/journal.pone.0136376)
Supplement: S1 Table — Hydrophobicity classes are define as follows: hydrophilic compounds (NSO:C ≥ 0.49 and molecular mass < 850 Da), moderately hydrophobic compounds (0.1 < NSO:C < 0.49 and molecular mass < 850 Da), and hydrophobic compounds (NSO:C ≤ 0.1 or molecular mass > 850 Da). (PDF) [file pone.0136376.s009.pdf]

S1 Table. Bulk chemical features of samples and controls. Hydrophobicity classes are define as follows: hydrophilic compounds (NSO:C ≥ 0.49 and molecular mass < 850 Da), moderately hydrophobic compounds (0.1 < NSO:C < 0.49 and molecular mass < 850 Da), and hydrophobic compounds (NSO:C ≤ 0.1 or molecular mass > 850 Da).

	Total peaks	% Formulas assigned	% Hydrophilic	% Moderately hydrophobic	% Hydrophobic
Marlin oil	11671	86	9	31	60
DCM 1					
Seawater control	6411	87	32	65	3
WAF	9679	93	14	58	27
WSF	10699	89	37	58	5
Seawater control (VSWE)	6174	88	31	65	3
WAF (lipid removed)	10240	94	17	66	17
WSF (lipid removed)	10648	89	40	56	4
DCM 2					
Seawater control	4357	89	28	69	3
WAF	7144	92	23	75	1
WSF	7648	86	43	54	3
Seawater control (VSWE)	5288	82	31	67	2
WAF (VSWE)	7448	94	28	71	1
WSF (VSWE)	10065	86	44	52	3
DCM-PPL					
Seawater control	1132	79	29	67	4
WAF	779	53	36	63	1
WSF	1495	63	35	64	1
Seawater control (VSWE)	2040	93	44	53	3
WAF (VSWE)	1496	82	30	69	1
WSF (VSWE)	1754	89	49	51	0
Seawater controls; PPL extraction only					
Seawater control	2185	90	51	46	3
Seawater control (VSWE)	1871	92	42	55	3
