# Supplementary material for: Chemical Composition and Potential Environmental Impacts of Water-Soluble Polar Crude Oil Components Inferred from ESI FT-ICR MS
Source: PLoS One. 2015 Sep 1;10(9):e0136376. doi: 10.1371/journal.pone.0136376 (PMC4556654; doi:10.1371/journal.pone.0136376)
Supplement: S2 Table — (PDF) [file pone.0136376.s010.pdf]

S2 Table. Heteroatom classes with relative abundance > 1% in the seawater-oil mixed samples.

	WAF	WSF	WAF (VSWE)	WSF (VSWE)
DCM 1	O_3 to O_6<br>NO_2 to NO_6<br>$N_{>1}O_{>1}$<br>SO_4, SO_5	O_3, O_4, $O_{>10}$<br>NO_2, NO_7,<br>$N_{>1}O$, $N_{>1}O_{>1}$<br>$S_{>1}O_{>1}$	O_3 to O_7<br>NO_2 to NO_7<br>$N_{>1}O_{>1}$<br>SO_4	O_3, $O_{>10}$<br>NO_7, $N_{>1}O$<br>$N_{>1}O_{>1}$<br>$S_{>1}O_{>1}$
DCM 2	O_3 to O_8<br>NO_3 to NO_8<br>$N_{>1}O_{>1}$<br>SO_4, SO_5	O_3 to O_5<br>NO_3, NO_4, NO_7<br>$N_{>1}O$, $N_{>1}O_{>1}$;<br>SO_4, SO_5,<br>$S_{>1}O_{>1}$	O_3 to O_9<br>$O_{>10}$<br>NO_3 to NO_9<br>$N_{>1}O$, $N_{>1}O_{>1}$<br>SO_4	O_3 to O_5<br>NO_3<br>$N_{>1}O$, $N_{>1}O_{>1}$<br>$S_{>1}O_{>1}$
DCM-PPL	SO_3 to SO_5<br>$S_{>1}O_{>1}$	SO_3 to SO_5<br>$S_{>1}O_{>1}$	O_4 to O_6, $O_{>10}$<br>$N_{>1}O_{>1}$<br>SO_3 to SO_5<br>$S_{>1}O_{>1}$	O_4 to O_6, $O_{>10}$<br>$N_{>1}O_{>1}$<br>SO_3 to SO_5
