# Supplementary material for: Chemical Composition and Potential Environmental Impacts of Water-Soluble Polar Crude Oil Components Inferred from ESI FT-ICR MS
Source: PLoS One. 2015 Sep 1;10(9):e0136376. doi: 10.1371/journal.pone.0136376 (PMC4556654; doi:10.1371/journal.pone.0136376)
Supplement: S3 Table — The coefficient values are obtained from the EPA EPI Suite. (PDF) [file pone.0136376.s011.pdf]

S3 Table. Selected fragment contribution coefficients for functional groups likely present in crude oil and petroleum-derived products. The coefficient values are obtained from the EPA EPI Suite [1].

Functional group	Example compound	Fragment contribution coefficient
Aromatic C-OOH	Carboxylic acids	-0.1186
Aromatic C-OH	Naphthol	-0.4802
N in 5-member ring	Carbazole	-0.5262
Aromatic C-NO_2	1-nitropyrene	-0.1823
S in 5-member ring	Dibenzothiophene	0.4082
Aromatic C-SO_3H	-	-3.16
