# Supplementary material for: Chemical Composition and Potential Environmental Impacts of Water-Soluble Polar Crude Oil Components Inferred from ESI FT-ICR MS
Source: PLoS One. 2015 Sep 1;10(9):e0136376. doi: 10.1371/journal.pone.0136376 (PMC4556654; doi:10.1371/journal.pone.0136376)
Supplement: S1 Text — (PDF) [file pone.0136376.s012.pdf]

# Chemical composition and potential environmental impacts of water-soluble polar crude oil components inferred from ESI FT-ICR-MS

# Supporting Information

*Yina Liu[1]*, Elizabeth B. Kujawinski[1]*

[1]Department of Marine Chemistry & Geochemistry, Woods Hole Oceanographic Institution, Woods Hole, Massachusetts 02543, United States

* Corresponding author Email: yina.liu@whoi.edu.

# Log $K_{ow}$ Estimation Method

We used the estimated octanol-water partition coefficients (log $K_{ow}$) of a series of pre-selected chemical structures to guide our further data interpretation. A total of 214 heteroatom-containing organic compounds were selected for estimating relationships between log $K_{ow}$ and NSO:C (see examples in S4 Fig.). Log $K_{ow}$ values and corresponding water solubility ($S$) values were estimated using the Estimation Program Interface (EPI) Suite obtained from the United States Environmental Protection Agency (EPA) (http://www.epa.gov/opptintr/exposure/pubs/episuite.htm) [1]. The KOWWIN package in the EPI suite estimated log $K_{ow}$ based on the atom/fragment contribution method [2]. For example, 1-nitropyrene ($C_{16}H_9NO$) has 16 aromatic carbons and 1 nitro group fragment. Therefore, its log $K_{ow}$ can be calculated as follows:

$$\log K_{ow\ \text{1-nitropyrene}} = 16 \times \text{A} + 1 \times \text{B} + 0.2290 \quad (1)$$

where A is the coefficient for an aromatic carbon (0.2940) and B is the coefficient for a nitro group (-0.1823) (see S3 Table). Hence, the log $K_{ow}$ for 1-nitropyrene is 4.75. Experimentally determined log $K_{ow}$ values were used when available from the EPI suite.

As shown above, heteroatom-containing functional groups tend to contribute negatively to the log $K_{ow}$ calculation. A lower log $K_{ow}$ corresponds to higher aqueous solubility. A significant correlation between log $K_{ow}$ and NSO:C was observed (Pearson's $r = -0.81$, $p < 0.001$; S6 Fig.). Therefore, high NSO:C is expected to yield lower log $K_{ow}$. A linear regression relationship can be developed within the NSO:C range considered in this study (2).

$$\log K_{ow} = 5.81 - 9.48(\text{NSO:C}) \quad (2)$$

# REFERENCES

1. U.S. EPA. Estimation Programs Interface Suite™ for Microsoft® Windows v 4.11. Washington, DC, USA.: United States Environmental Protection Agency; 2012.
2. Meylan WM, Howard PH. Atom/fragment contribution method for estimating octanol–water partition coefficients. J Pharm Sci. 1995;84(1):83-92.
3. McKenna AM, Nelson RK, Reddy CM, Savory JJ, Kaiser NK, Fitzsimmons JE, et al. Expansion of the analytical window for oil spill characterization by ultrahigh resolution mass spectrometry: Beyond gas chromatography. Environ Sci Technol. 2013;47(13):7530-9.
